# Mental Hospitals and the Insurance Act

**Published:** 1912-08-24

**Authors:** 


### The Hospital

The Workers' Newspaper of Administrative Medicine and Institutional Life, Administration, National Insurance and Health.

No. 1364, Vol. LII. SATURDAY, AUGUST 24, 1912. PRICE ONE PENNY.

### MENTAL HOSPITALS AND THE INSURANCE ACT.

In all mental hospitals the indoor staff receive medical attendance during sickness. It is also the general custom to pay wages during that period. This is not done as an act of grace on the part of the employer. It is part of the remuneration given to the staff for services rendered. The fact that they are thus treated during illness is taken into consideration when the question of an increase in salary is discussed. The attendants and nursing staff of a mental hospital, therefore, are already buying the greater part of the benefits of the Insurance Act, and are paying for them by their work. Practically the only benefits they do not receive at present under the terms of their engagement are sanatorium benefit and the five shillings weekly disablement allowance. Under the Asylum Officers' Superannuation Act they can, after ten years' service, receive a small pension proportionate to the number of years served. It would take the average nurse twelve years to earn a pension of five shillings weekly. The fact that a pension is obtainable was also taken into account when the amount of pecuniary reward for the nurse's services was fixed. Towards this pension a deduction of 3 per cent. on the wages and value of emoluments is made. There is, however, a period of at least ten years during which, under the terms of their engagement, no provision is made for any illness which entirely disables the member of the staff from work, except that under such circumstances the contributions paid under the Superannuation Act can be claimed back. The Insurance Act provides five shillings weekly disablement allowance during that period. It is, however, the best ten years of picked lives, and the risk is not a great one.

The nursing staff of a mental hospital and the local authority which employs them are alike anxious to be excepted from the operation of the Insurance Act. The local authority desires this because it is more economical and because it is more convenient. It is less expensive to give medical attendance and pay full wages during the whole period of sickness than to bring the staff under the operation of the Act. Even if this were not so, and the local authority wished to take advantage of the Act, it is faced with the difficulty that members of the staff who are sick must of necessity be treated and nursed in the institution. The vast majority of them do not come from the neighbourhood of the mental hospital in which they are employed, and when ill would have nowhere to go except to the workhouse infirmary. Some would be too ill to be moved if so inhuman a course were even thought practicable. There are obvious administrative difficulties in the way of having outside medical men coming into the institution to treat sick members of the staff. The authority as employer would obtain no relief from its present custom as regards the care of the sick if the staff were under the Insurance Act. Therefore, as an economy, and almost as a necessity, the local authority wishes for exception. To the staff also the course appears desirable. They are already purchasing medical attendance and the payment of wages during sickness. They do not think sanatorium benefit and five shillings weekly disablement allowance during the best ten years of picked lives a fair return for the contributions under the Act. Their position is different from that of the ordinary artisan. The latter receives payment for services entirely in cash. Out of the money received he has hitherto provided for sickness. In future the State and his employer are going to bear five-ninths of this expense. He practically receives an increase of wages to that amount. Why the artisan who earns forty shillings weekly should be subsidised by the State in order that he may obtain his medicine cheap is hard to understand, though doubtless there were political exigencies which rendered it expedient to give unto him who hath. The staff of the mental hospital are not so fortunate. Even if excepted and not called upon to contribute, they will in the future in practice only receive what they were in the past paying for by their labour. The State makes no contribution towards provision for their sickness; the employer makes none, because the conditions under which exception is granted by the Insurance Commissioners do not materially differ from those under which the staff worked before the passing of the Insurance Act. The Insurance Commissioners appear to think that the same principle should extend to provision for disablement. The wages of those who work among mental patients were fixed with a due regard to the fact that pensions were granted for faithful service. In order to assure those pensions the Superannuation Act made a deduction of 3 per cent. from the salary and value of emoluments compulsory. In the event of enforced retirement through illness before ten years have been served, a member of the staff of a mental hospital is entitled under the Superannuation Act to a return of the amount deducted towards pension. This right, which previously existed, is now considered a sufficient provision in case of disablement, and also as making provision for those leaving the employment. In the latter case no money is returnable if the reason for leaving be voluntary resignation. In any event, the staff only receive their own contributions back, and the full provision is thrown entirely upon them. In effect it means that if a woman after three years' service contracts tubercle she would leave with about four pounds wherewith to provide sanatorium, medical attendance, and everything required. Why should the State be relieved entirely from its obligation to provide two-ninths of the money contributed? At one time it appeared as though the Insurance Commissioners thought that something further should be done. It may be that there are legal difficulties in the way of local authorities making provision for permanent disablement. As matters stand, the members of the staff of mental hospitals are not, under the Insurance Act, receiving benefits equal to those of the ordinary manual labourer.

